# *Mycoplasma pneumoniae* triggers pneumonia epidemic in autumn and winter in Beijing: a multicentre, population-based epidemiological study between 2015 and 2020

**DOI:** 10.1080/22221751.2022.2078228

**Published:** 2022-06-02

**Authors:** Xue Wang, Maozhong Li, Ming Luo, Qin Luo, Lu Kang, Hui Xie, Yiting Wang, Xiali Yu, Aihua Li, Mei Dong, Fang Huang, Cheng Gong

**Affiliations:** a Beijing Center for Disease Prevention and Control, Institute for Immunization and Prevention, Beijing, People’s Republic of China; b College of Public Health, Capital Medical University, Beijing, People’s Republic of China

**Keywords:** *Mycoplasma pneumoniae*, surveillance, macrolide-resistant *Mycoplasma pneumoniae*, *Mycoplasma pneumoniae* pneumonia, acute respiratory tract infection

## Abstract

The objective of this paper is to explore the characteristics of *Mycoplasma pneumoniae* (MP) epidemics in Beijing, China. Patients with acute respiratory tract infection (ARTI) were enrolled from 35 sentinel hospitals in Beijing, 2015–2020. Their medical records were reviewed and respiratory specimens were collected for assay for nucleic acids of 24 respiratory pathogens, including MP. The genotypes of MP were analysed using a real-time PCR method. The domain V of 23s rRNA gene was sequenced to identify macrolide-resistant mutations. A total of 41,677 specimens of ARTI patients were included, with an MP positive rate of 6.16%. MP prevalence mainly occurred between August and January, and peaked in October. The increase in the MP detection rate was coincident with the elevation of the reported number of patients with pneumonia in the 35 sentinel hospitals. One or more respiratory pathogens were co-detected in 27.1% of the MP-positive patients. Type 1 MP remained predominant, and the macrolide-resistant rate of MP had exceeded over 90%. A2063G mutation accounted for 99.0% of macrolide-resistant MP infections. MP epidemic in Beijing mainly occurred between August and January with a remarkable high macrolide-resistant rate. MP is one of the important contributors to the pneumonia epidemic in autumn and winter in Beijing.

## Introduction

*Mycoplasma pneumoniae* (MP) infection is often considered a self-limiting disease. Due to the relatively mild nature, the similarity in presentation to respiratory tract infections caused by other respiratory pathogens, and the lack of reliable point-of-care diagnostic tests to confirm the microbiological diagnosis, many sporadic MP infections and even outbreaks are likely to be undetected.

However, MP is one of the leading pathogens that cause human upper and lower respiratory tract infections. After world-spread use of pneumococcal conjugate vaccine (PCV13), MP has become the most common pathogen of community-acquired bacterial pneumonia in children [1] and accounts for 10–40% of cases of community-acquired pneumonia (CAP) [2,3]. Among European adults, the incidence of MP pneumonia is 4–8% during endemics and as high as 20–40% during epidemics [4,5]. In a report by the multinational Asian Surveillance Network for Drug-Resistant Pathogens (ANSORP), MP accounts for 11% of adults with CAP in eight Asian countries [6]. The positive detection rate of MP reaches 10–30% in CAP children in China [7]. In addition to respiratory tract infections, MP probably induces extrapulmonary damage, such as diarrhoea, inflammation of the central nervous system, acute myocardial injury, arthritis, or otitis media [2,8–10]. These extrapulmonary illnesses are a result of MP infection itself and/or an excessive immune response or inflammation triggered by the “superior antigen” of MP, which can eventually develop into a life-threatening disease [11–13].

Macrolides have been used as first-line antibiotics for the treatment of MP infection for several decades [14]. However, since pneumonia caused by macrolide-resistant MP (MRMP) was first reported in Japan in 2000, the prevalence of MRMP has been increasing due to the extensive application of macrolide antibiotics [15–18]. There are significant differences in the prevalence of MRMP among different countries or regions in the world. Namely, the prevalence of MRMP remains under 30% [19,20] in the United States and Europe. In contrast, countries or regions in Asia have a significantly higher prevalence, which exceeds 80% in Japan [21] and even reaches 90% in China [14,22,23]. Considering the emergence and epidemic of MRMP, it is more challenging for the administration of MP infection, particularly for the infections among children and teenagers [24].

A worldwide organized surveillance program similar to influenza surveillance has not been initiated for MP infection. Presently, the few studies on the characteristics of MP epidemics in Beijing are mostly based on one or several clinical settings [22,23], and covered only a small subset of the population with MP infection [25–27]. Namely, these studies only included CAP and excluded upper respiratory tract infections, or included children and excluded adults, which is why it is difficult to assess the true impact of this pathogen on public health. This study was based on the Beijing Respiratory Pathogen Surveillance System (RPSS), which intended to cover a more complete illness profile of respiratory infections caused by MP (including acute upper respiratory tract infection, non-severe pneumonia, and severe pneumonia), and included patients of all age categories who presented at or were admitted to 35 sentinel hospitals in Beijing. Thus we conducted a systematic exploration of the prevalence of MP to obtain the comprehensive insight into the characteristics of MP epidemics in Beijing.

## Materials and methods

### Ethics statement

The protocol of this study was approved by the Ethics Committee of the Beijing Center for Disease Prevention and Control (BJCDC). A written informed consent was obtained from each of the included patients or their legal guardians.

### Study population and specimen collection

Based on the RPSS, patients with acute respiratory tract infection (ARTI) were enrolled from 35 representative sentinel hospitals of different kinds and levels in Beijing in the period from January 1, 2015, to December 31, 2020 (see Supplementary table 1 in appendix supplementary). Approximately 5 patients with upper respiratory tract infection (URTI) and 15 patients with CAP were required to be recruited from each sentinel hospital per month.

URTI patients were included if they presented with fever and/or respiratory symptoms, e.g. cough, sputum production, and sore throat. CAP patients were included if they had evidence of CAP according to the guidelines for the diagnosis and treatment of adult community-acquired pneumonia in China (2016 edition) and the guidelines for the management of community-acquired pneumonia in children (the revised edition of 2013) [28–30]. They were further classified as severe CAP (SCAP) and non-severe CAP (NSCAP) based on illness severity according to the criteria described previously [31]. The inclusion and exclusion criteria of the study subjects were described in detail in appendix supplementary.

All of the cases were investigated by their physicians after enrolment using a uniform questionnaire, which mainly included demographic data, epidemiological data, clinical manifestations, radiographical imaging data, and laboratory data. Respiratory specimens were collected as early as possible (at the time of presentation for outpatients or within 3 days since admission for inpatients) and included at least one of the following types of specimens: pharyngeal swabs, nasopharyngeal swabs, nasopharyngeal aspirates, sputum, pleural effusion, tracheal aspirates, or bronchoalveolar lavage fluid.

In addition, the numbers of URTI patients and CAP patients were reported weekly by the 35 sentinel hospitals from January 1, 2017, to December 31, 2020.

### Laboratory assay

Nucleic acid test was performed on the collected clinical specimens for nine respiratory viruses, MP and *Chlamydia pneumoniae* (CP) (see Appendix supplementary). Moreover, the specimens obtained from lower respiratory tract, a subset of the total specimens, were tested for a panel of 13 respiratory bacteria (see Appendix supplementary).

MP-positive specimens were further genotyped into types 1 and 2 with a real-time PCR method described by Zhao et al. [32]. The domain V of their 23s rRNA gene was sequenced to identify the macrolide-resistance mutation [16].

### Statistical analysis

Continuous variables were presented as mean (95% CI) or median (interquartile range, IQR), and categorical variables were presented as numbers (%). Comparisons between different groups were conducted by the χ² test or Fisher’s exact test. The restricted cubic spline (RCS) method was used to analyse the relationship between the probability of MP infection and age using R language 4.0.5 and RMS software package. Interaction between multiple respiratory pathogens was analysed by multiple-stage logistic regression. A two-sided *P*-value lower than 0.05 was considered statistically significant. Statistical analysis, except for RCS analysis, was done using SPSS 19.0 (SPSS Inc., Chicago, IL, USA).

## Results

### Study population

From January 1, 2015, to December 31, 2020, a total of 41,677 specimens of patients with ARTI were included in this study, including 14,099 specimens of patients with URTI, 22,125 specimens of patients with SNCAP, and 5453 specimens of patients with SCAP. The mean number of specimens enrolled per month was 624 (95% CI, 605–644) between 2015 and 2019, which was reduced to 351 cases per month due to the COVID-19 pandemic in 2020. Male patients accounted for 55.9%, which is in line with the population structure in China. The median age was 37 years (P25, 9 years; P75, 67 years). Among the included patients, 2566 (6.16%) were confirmed as MP infections.

### Seasonality

**Figure 1.**
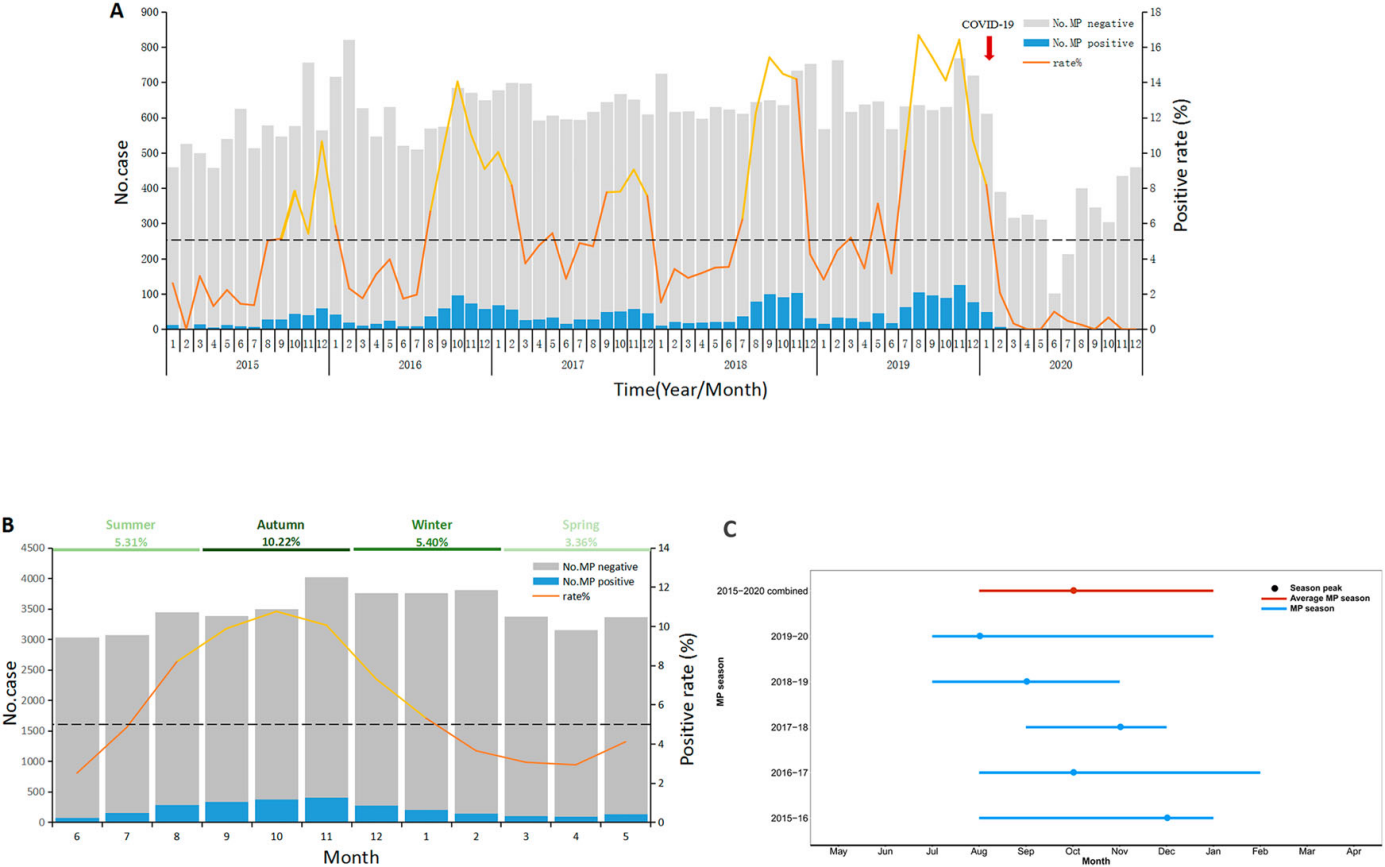
MP prevalence among all-aged patients with acute respiratory tract infection in Beijing, China, from January 1, 2015, to December 31, 2020. (A) The number of patients with acute respiratory tract infection and MP positive rates by month. (B) Average percentage of positive detections for MP among all aged patients with acute respiratory tract infections per month. (C) MP season duration and peak by year. The grey bars (panels A and B) denote the number of MP-negative cases. The blue bars (panels A and B) represent the MP-positive cases. The solid lines (panels A and B) indicate the percentage of MP-positive cases among the total cases included in this study, where the orange part represents the non-epidemic period and the yellow part represents the popular period. The dash lines (panels A and B) denote the threshold of MP-positive rate (5%) assigned in this study. The blue horizontal lines (panel C) indicate the MP season duration, and the black dot represents the MP epidemic peak. MP, *Mycoplasma pneumoniae*.

From 2015 to 2020, the prevalence of MP in patients with ARTI in Beijing exhibited a distinct one-peak-one-year epidemic pattern. The yearly positive rates (from 2015 to 2020) of MP among patients with ARTI were 3.97%, 6.12%, 6.46%, 7.12%, 9.30%, and 1.52%. Hence, after an increasing trend from 2015 to 2019, the rate fell to the lowest level in 2020 (Figure 1A).

The positive rates of MP among patients with ARTI per month increased significantly from August, peaked in October, and fell down in January of next year. The epidemics of MP lasted for about 6 months (Figure 1B).

In this study, the MP epidemic season was defined as the consecutive months in which the PCR positive rate of MP was continuously over 5%, and the cases in the epidemic season accounted for 74% of the total cases (Figure 1C).

In some of the examined years, the onset of the epidemic season was advanced or delayed by 1–2 months. In 2018–2019 and 2019–2020, the onset of the epidemic season was advanced to July, while in 2017–2018, the onset of the epidemic was delayed to September. The epidemic season lasted for 6, 4, and 5 months in 2015–2016, 2017–2018, and 2018–2019, respectively, while it was extended to 7 months in 2016–2017 and 2018–2019 (Figure 1D).

### Positive rates of MP by age group and severity of illness

**Figure 2.**
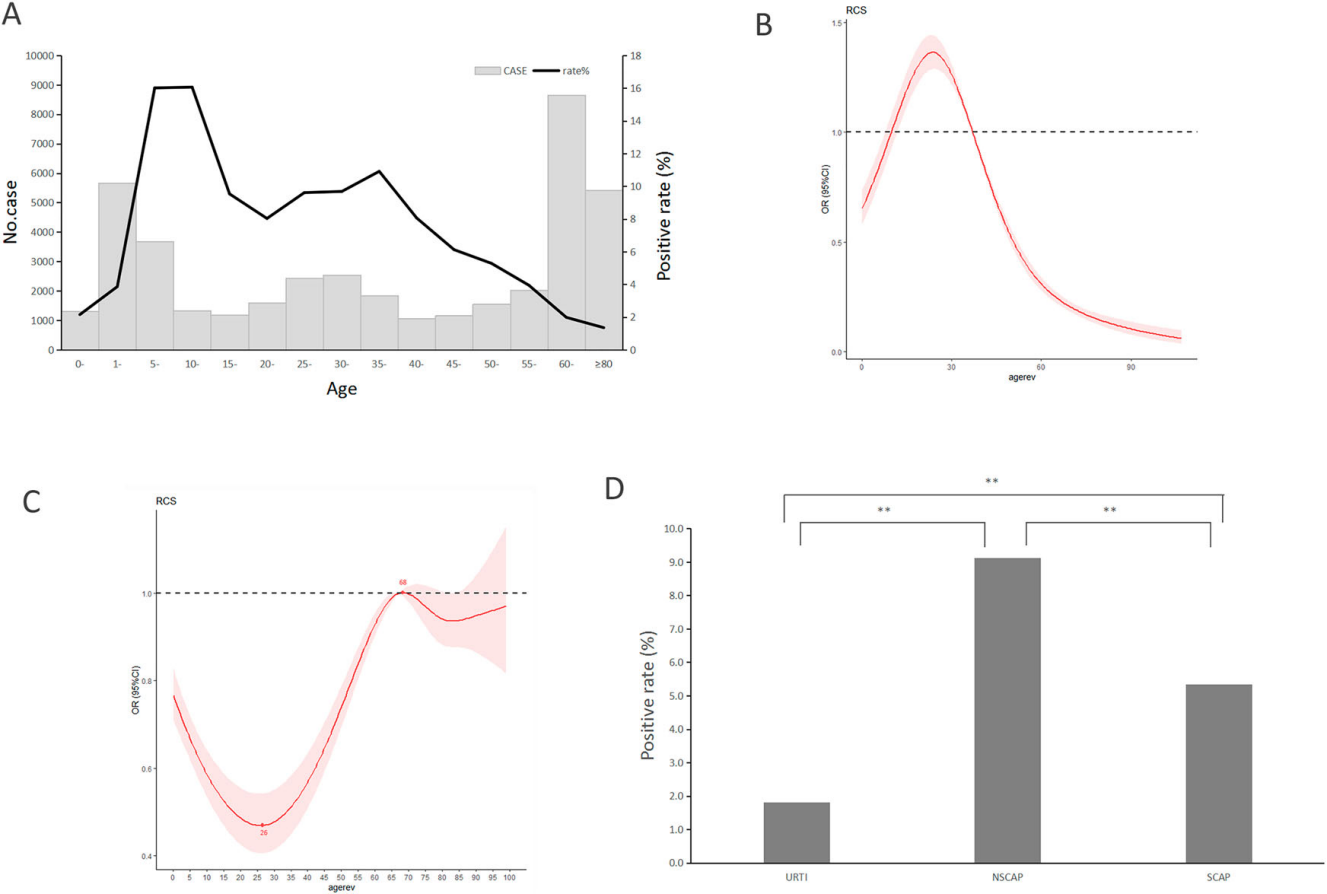
MP prevalence among patients with acute respiratory tract infections (ARTI) in Beijing, China, from January 1, 2015, to December 31, 2020, by age, or by sex, or by severity of illness. (A) MP infections among patients with ARTI by age. The light grey bars denote the total number of ARTI cases included in this study. The dark black line indicates the percentage of MP-positive cases among total cases. (B) Analysis of nonlinear influence of age on the risk of MP infection (*P* < 0.001). (C) Analysis of nonlinear influence of age on the risk of MP infection among patients with SCAP. (D) MP infections among patients with ARTI by severity of illness. The rug plot (panel B and C) along the *x*-axis shows the observed values; grey shading indicates 95% CIs. The dark black bars (panel D) indicate the percentage of MP-positive cases among total cases.

The positive rate of MP among patients aged 5–44 years was significantly higher than that in other age groups (*χ*^2^ = 1655.765, *P* < 0.001), with a peak in 10–14-year-old group (16.06%) (Figure 2A). The median age of 2566 patients with confirmed MP infection was 24 years (P25, 8 years; P75, 37 years).

There was a nonlinear correlation between the probability of MP infection and age. For patients under 24 years of age, the probability of MP infection increased with age, while for patients aged 24 years and older, the probability of MP infection decreased as age increased (Figure 2B). Regarding patients with SCAP, there was also a nonlinear correlation between the probability of MP infection and age, but the changing trend was almost opposite to that in overall ARTI patients. Namely, the probability of MP infection decreased with age among the SCAP patients under 26 years of age, then increased gradually as age increased until 68 years, and finally fell down again (Figure 2C).

The positive rate of MP in male patients (5.69%, 1324/23286) was lower than that in female patients (6.75%, 1242/18391) (*χ*^2^ = 20.266, *P* < 0.001). Notably, the positive rate of MP among patients with NSCAP (9.12%, 2018/22125) was significantly higher than that among patients with URTI (1.82%, 256/14099) (*χ*^2^ = 781.086, *P* < 0.0125) or SCAP (5.35%, 292/5453) (*χ*^2^ = 80.848, *P* < 0.0125) (Figure 2D).

### Pathogens co-detected with MP

Nucleic acids of respiratory pathogens other than MP were detected in specimens of 27.1% (695/2566) of the patients infected with MP, including lower respiratory tract specimens (sputum, tracheal aspirates, bronchoalveolar lavage fluid) of 564 patients and upper respiratory tract specimens (pharyngeal swabs) of 131 patients(Table 1). The most common co-detected pathogen was *S. pneumoniae* (240 cases) followed by *H. influenzae* (137 cases), *S. maltophilia* (80 cases), and *P. aeruginosa* (77 cases).

**Table 1.** Respiratory pathogens co-detected with MP.

Specimen	Number of co-detected pathogens					Total	No. specimens	Detection rate, %
	1	2	3	4	5			
Obtained from URT	121	8	2	–	–	131	1244	10.5
Pharyngeal swabs	121	8	2	–	–	131	1244	10.5
Obtained from LRT	368	128	42	17	9	564	1322	42.7
BALF	54	15			1	70	217	32.3
Tracheal aspirates	1			1		2	4	50
Sputum	313	113	42	16	8	492	1101	44.7
Total	489	136	44	17	9	695	2566	27.1

URT, upper respiratory tract; LRT, lower respiratory tract; BALF, bronchoalveolar lavage fluid.

**Figure 3.**
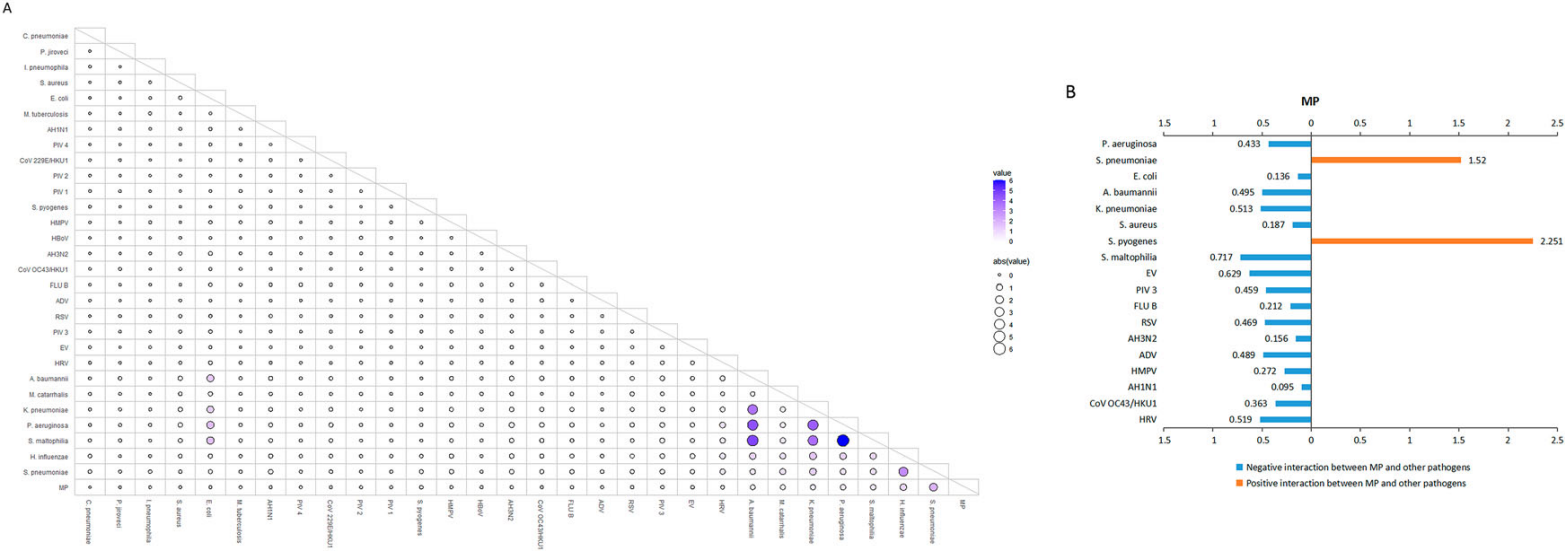
Co-detected pattern and interactions of MP and other respiratory pathogens in patients with acute respiratory tract infection in Beijing, China, from January 1, 2015, to December 31, 2020. (A) Co-detected pattern of MP and other respiratory pathogens. Co-detected rates were calculated pairwise. A total of 30 indicators were included (15 respiratory virus-related, 13 respiratory bacteria-related and MP, *Chlamydia pneumoniae;* except inﬂuenza virus A). For pathogens “X” and “Y”, the numerator was the number of patients in whom “X” and “Y” were co-detected, and the denominator was the total number of patients who were both tested “X” and “Y”. Bigger size and darker colour of the circles indicate higher co-detected rates between two pathogens. (B) The interactions among MP and other pathogens were estimated by host-scale logistic regressions. Positive interactions with two-sided *P*-value <0.05 are denoted with orange bars and the negative interactions with two-sided *P*-value <0.05 are denoted with blue bars. The *P* values were not adjusted for multiple comparisons. The length of the coloured bars and the adjacent number indicate the odds ratio (OR) of the interaction. The interaction was determined as significant both without adjusting for multiple pathogens and with adjusting for multiple pathogens. Influenza virus AH1N1 2009 pandemic and AH3N2 (AH1N1, AH3N2), inﬂuenza virus B (FLU B), respiratory syncytial virus (RSV), parainﬂuenza virus 1, 2, 3, and 4 (PIV 1, 2, 3, 4), adenovirus (AdV), human rhinovirus (HRV), human metapneumovirus (HMPV), human coronavirus 229E/NL63, OC43/HKU1 (CoV 229E/NL63, OC43/HKU1), human bocavirus (HBoV), human enterovirus (EV).

The most frequent co-detected pattern was observed between MP and *S. pneumoniae* (2.01%), followed by that between MP and *H. influenzae* (1.15%), MP and *S. maltophilia* (0.67%), and MP and *P. aeruginosa* (0.64%) (Figure 3A). Most of the interactions referring to MP were negative, predominantly occurring between MP and all 10 viruses, and between MP and 6 bacteria, while positive interaction was identified only between MP and 2 bacteria (*S. pneumoniae*, *S. pyogenes*) (Figure 3B).

### Contribution of MP infection to the prevalence of CAP in Beijing

A total of 2,290,510 patients with ARTI were reported in Beijing in this study, including 987,045 cases of URTI and 1,303,465 CAP cases. The median number of the reported cases per month was 9265 (P25, 7848 cases; P75, 12,476 cases).

**Figure 4.**
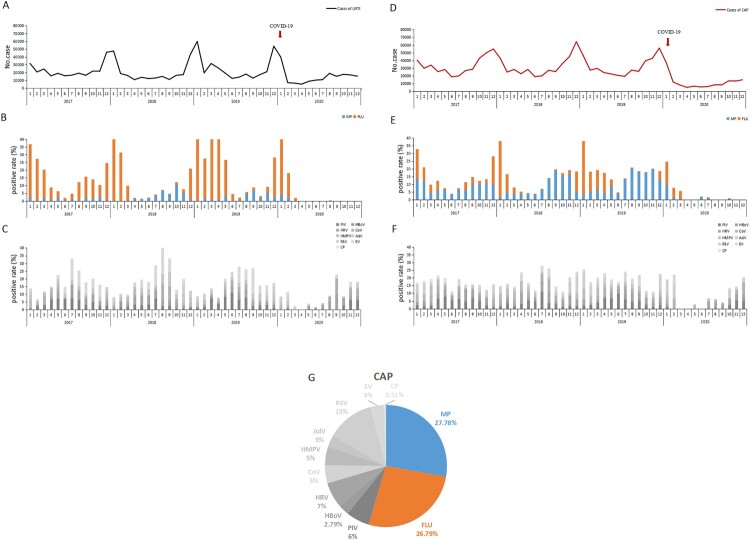
The reported numbers of patients with upper respiratory tract infection (URTI) or community-acquired pneumonia (CAP) presented to 35 sentinel hospitals in Beijing, China, from January 1, 2017, to December 31, 2020; the positive detection rates of each of 11 respiratory pathogens, including MP, by month. (A) The reported numbers of patients with URTI by month. (B) The positive rates of MP and influenza virus among patients with URTI by month. (C) The positive rates of nine respiratory viruses among patients with URTI by month. (D) The reported numbers of patients with CAP by month. (E) The positive rates of MP and CP among patients with CAP by month. (F) The positive rates of nine respiratory viruses among patients with CAP by month. (G) The pathogenic spectrum of CAPs in pneumonia epidemic season. The red arrow (panels A and D) denotes the time point of implementation of public health measures against the COVID-19 pandemic in Beijing. URTI, upper respiratory tract infection; CAP, community-acquired pneumonia.

The number of the reported cases per month of CAP always surged earlier by about 1 or 2 months than that of URTI cases. Namely, the number of CAP cases began to rise from October, reached the peak in December, and fell down in January, while the number of URTI cases rose dramatically in December, peaked in January, and then decreased in February (Figure 4).

Referring to the positive detection rates of 11 common respiratory pathogens, the number of the reported URTI cases per month approximately rose and fell at the same time as the positive detection rate of influenza virus among URTI cases (Figure 4A–C). The elevation in the reported number of the reported CAP cases per month initiated in the period during which the positive detection rate of MP among CAP cases increased dramatically. However, the detection of the other respiratory pathogens (including influenza virus) remained at a low level in the same period. As the positive detection rate of influenza virus among CAP cases rose, this increasing trend in the number of CAP cases was further enhanced. Generally speaking, the total detection of MP and influenza virus fluctuated approximately with the number of CAP case reported, while the total detection of the other respiratory pathogens independently remained at an almost constant level in the same period (Figure 4D–F).

During the pneumonia epidemic season between October and January, MP and influenza virus accounted for 27.78% and 26.79% of CAP, respectively (Figure 4G).

### The alteration of genotype

A total of 866 specimens of MP-positive patients from 2018 to 2020 were used for the analysis of the P1 gene type in this study. Type 1 accounted for 62.0% cases in 2018, 60% in 2019, and increased significantly to 83.7% in 2020 (Table 2).

**Table 2.** Genotype characteristics and macrolide-resistance mutations in domain V of 23 rRNA gene of MP in Beijing from 2018 to 2020.

Year	Mutation in the 23S rRNA	Genotype number			Total
		Type 1	Type 2	Mix	
2018	A2063G	95	35	0	130
	A2064G	1	0	0	1
	None	4	41	0	45
	-	166	87	0	253
2019	A2063G	155	80	3	238
	A2064G	0	1	0	1
	A2063C	0	1	0	1
	None	4	20	1	25
	-	74	48	1	123
2020	A2063G	26	4	0	30
	A2063C	1	0	0	1
	None	1	1	0	2
	-	13	3	0	16
Total		540	321	5	866

#### MRMP

The total proportion of MRMP in Beijing from 2018 to 2020 was 84.72% (402/474), with 74.43% in 2018, 90.57% in 2019, and 93.94% in 2020. The proportion of type 1 MRMP remained above 95% from 2018 to 2020, while that of type 2 MRMP increased gradually from 2018 to 2020 and reached over 80% in 2020.

Three kinds of point mutations in domain V of 23s rRNA were detected: A2063G, A2064G, and A2063C. Of these mutations, A2063G accounted for 99.0% (398/402), followed by A2064G (0.50%, 2/402) and A2063C (0.50%, 2/402). No macrolide-resistant mutations at locus 2617 were detected. A2063G was the dominant mutation in both type 1 and type 2 MRMP, accounting for 96.17% and 98.35%, respectively (Table 2).

## Discussion

This study showed that MP infection in Beijing occurred throughout the year. A higher prevalence was between August and January, showing that MP infection exhibited a one-peak-one-year epidemic pattern. Several studies have reported that MP epidemic surges every 3–7 years probably due to antigenic shift or decreased herd immunity [33]. Among five consecutive MP seasons, we observed that MP epidemic in Beijing peaked in 2016, followed by that in 2019. However, a similar interval between two adjacent epidemics was not observed in this study due to a short surveillance period.

In this study, we observed that the positive rates of P1 adhesin gene-based nucleic acid test for MP were always over 6.16% during the five consecutive MP seasons, and we assumed that the epidemic season of MP occurred if the PCR positive rate of MP among ARTI patients exceeded 5% per month in Beijing. This threshold contributed to the timely identification of MP epidemic season and the comparability of different studies. Of course, the MP prevalence intensity varies even between different regions of China, so a local value of the threshold of MP detection probably needs an adjustment according to the characteristics of MP prevalence in a particular region.

Public health measures against COVID-19, such as wearing masks in public places, keeping social distance, prohibiting large assemblies, and isolating and quarantining patients and close contacts as early as possible, were implemented in Beijing since January 23, 2020. These measures were confirmed to effectively control or even exterminate COVID-19 epidemic in Beijing. Meanwhile, they also dramatically reduced the prevalence of multiple respiratory pathogens, including MP [34]. Although the role that each measure played in the course of preventing epidemics of these respiratory pathogens is unclear, they, as a whole, were shown to effectively stop or slow down the spread of these respiratory pathogens, including MP, in the population. This is also the first time that the several-decade epidemic pattern of MP in Beijing was altered completely by artificial interventions.

We observed a nonlinear correlation between the risk of MP infection and age of patients with ARTI. Among individuals aged under 24 years, the risk of MP infection increased with age, while among those aged 24 years and older, the risk of MP infection decreased as age increased. We compared the MP positive rates among different age groups and found that the MP positive rate was higher in the 5–44-year-old group and peaked in the 10–14-year-old group, which is approximately consistent with a previous study that showed that MP infection occurred more often among children and teenagers aged 5–14 years [35]. The distribution of the MP detection rate by age was lower at both ends and higher in the middle, which was significantly different from many other respiratory pathogens (e.g. enterovirus, respiratory syncytial virus). The reason underlying this specific age distribution is not completely clear and may be associated with the expression of the receptor of MP on epithelial cells of the respiratory tract, and/or with host immune defence. Cilia of the epithelial cells in the respiratory tract are considered the initial attachment site for P1 cytadhesin in the course of MP infection [36]. Community-acquired respiratory distress syndrome (CARDS) toxin of MP is considered the most important virulence factor that presumably triggers cell pyroptosis and inflammatory injury via the NLRP3 signalling pathway [37]. According to Bajantri et al., the “common antigen” between MP and the host presumably induces excessive immune response and causes refractory MP infections [5]. The population aged 5–44 years possesses more mature cilia of epithelial cells, which facilitates the MP attachment and thereby increases the risk of MP infection. These individuals also have a more mature immune system, which contributes to maintaining the balance between immune protection and immune injury, thus preventing MP-infected host from developing a severe illness. In this study, we observed that the changing trend of MP infection probability with age among SCAP patients was almost opposite to that among total ARTI patients, which suggested that young children have a higher risk to develop severe illness even though they have lower probability of MP infection.

Clinical manifestations of pneumonia caused by MP are indistinguishable from those of pneumonia caused by other respiratory pathogens. In this study, in respiratory specimens of 27.1% (695/2566) of MP-positive patients, one or more other respiratory pathogens were co-detected. These co-detected pathogens may have been only planted in the respiratory tract or may have participated in the pathogenic process together with MP. Currently, as SARS-CoV-2 spreads around the world, a co-infection of MP and SARS-CoV-2 is of great concern. Xing et al. [38] reported that, among 68 COVID-19 cases, the MP detection rate among the confirmed COVID-19 patients was 23.3% in Qingdao city and 2.63% in Wuhan city. Given the COVID-19 pandemic, this kind of co-infection occurs more frequently and probably leads to more severe clinical outcomes; thus, we should pay more attention to it, particularly during the MP epidemic season.

Before the COVID-19 pandemic, influenza was the leading cause of acute respiratory infections. Influenza epidemics have also been considered the main cause for the dramatic elevation in the incidence of pneumonia in autumn and winter in Beijing. However, we observed that the onset time of pneumonia epidemic in Beijing was about 1 or 2 months earlier than that of the influenza epidemic, and was approximately coincident with that of MP epidemic, with no observed epidemics of other common respiratory pathogens in the same period (October and November). Moreover, MP accounted for 27.78% of CAPs in pneumonia epidemic season, and our previous study also reported that MP, after influenza virus, was the second most common pathogen of CAP in Beijing [29]. Taken together, the high prevalence of pneumonia in autumn and winter in Beijing was initiated by the MP epidemic, and then further increased and prolonged by the subsequent influenza epidemic. Thus influenza vaccination alone is not enough to significantly reduce the high prevalence of pneumonia in autumn and winter in Beijing, and some of the abovementioned measures confirmed effective against MP should also be taken into consideration.

**Figure 5.**
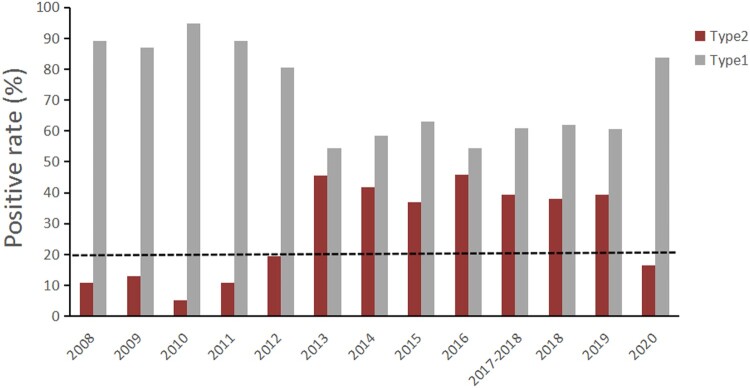
Time trend of the prevalence of two genotypes of MP, Beijing, China, 2008–2020. The grey bars denote the proportion of type 1 MP among total MP-positive cases, and the red bars denote type 2. The data on the prevalence of the two genotypes between 2008 and 2018, based on previous studies[1–3]. The black horizontal dash represents the threshold (20%) of the proportion of type 2.

**Figure 6.**
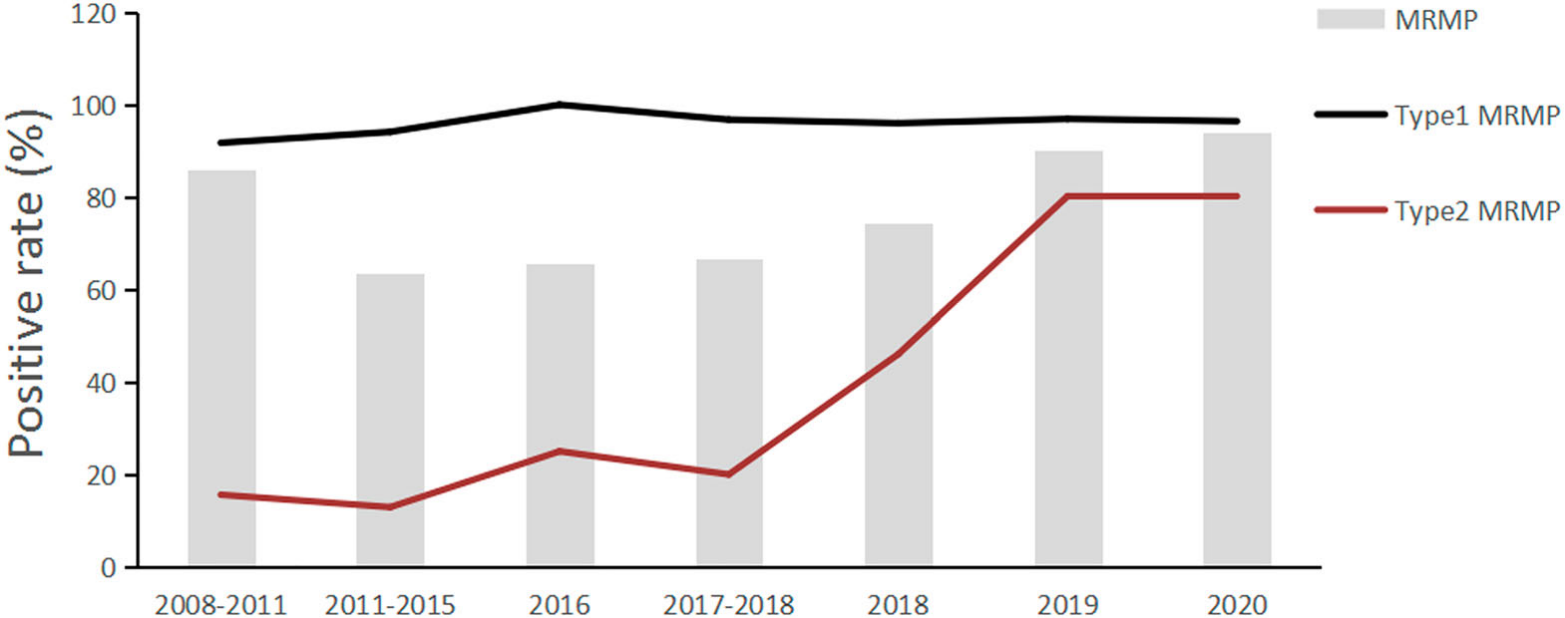
Time trend of MRMP prevalence in Beijing, China, 2008–2020. The grey bars indicate the overall proportion of MRMP among total MP-positive patients. The black line denotes the MRMP proportion among patients infected with type 1 MP, and the red line denotes that in patients infected by type 2 MP. The data on the MRMP prevalence in Beijing between 2008 and 2018 were retrieved from multiple previous studies[1–3]. MRMP, macrolide-resistant *Mycoplasma pneumoniae*.

Integrating our findings with those of previous studies [23,25,27,32], we found some distinct changes associated with MP infections in Beijing during the 13-year period from 2008 to 2020: The prevalence of type 2 MP increased significantly in 2013 and fell in 2020 (Figure 5). The prevalence of type 1 MRMP had been hovering at 90% from 2008 to 2020, while that of type 2 MRMP retained at a very low level until 2015, then surged rapidly since 2016 and exceeded 80% in 2020 (Figure 6). This alteration was probably associated with restriction on the use of tetracyclines and fluoroquinolones for treatment of children with MP infection since 2015 in China [39,40], which showed an urgent need for alternative antibiotics for children with MRMP infection in China.

In conclusion, MP prevalence exhibited a one-peak-one-year pattern in Beijing with an epidemic season that usually occurred between August and January with a peak in October, which was altered completely by public health measures against COVID 19 in 2020. Type 1 MP remained dominant while the prevalence of MRMP exceeded over 90%. Notably, it was MP, other than the influenza virus, that first triggered pneumonia epidemics in autumn and winter in Beijing each year. These facts should be considered when public health policies are made.

## Supplementary Material

Supplemental Material
