# Supplementary material for: Mycoplasma pneumoniae triggers pneumonia epidemic in autumn and winter in Beijing: a multicentre, population-based epidemiological study between 2015 and 2020
Source: Emerg Microbes Infect. 2022 Jun 2;11(1):1508–17. doi: 10.1080/22221751.2022.2078228 (PMC9176688; doi:10.1080/22221751.2022.2078228)
Supplement: Supplemental Material [file TEMI_A_2078228_SM4377.doc]

**Appendix supplementary**

1. **Sentinel hospitals**

Supplementary table 1. Thirty five sentinel hospitals from all of 16 districts in Beijing, China.

| Districts | Sentinel Hospitals |
| --- | --- |
| Dongcheng district | Tiantan hospital, Tongren hospital, Beijing No.6 hospital, Dongzhimen hospital, Beijing longfu hospital |
| Xicheng district | Peking university people's hospital, Beijing children' hospital, Jian gong hospital, Fuxing hospital |
| Chaoyang district | The first hospital of Qsinghua university, China-Japan friendish hospital, Chuiyangliu hospital, Beijing hospital of intergrated traditional chinese and western medicine, Anzhen hospital |
| Haidian district | Aerospace center hospital, Beijing Shijitan hospital, haidian hospital, Peking uinversity thrid hospital |
| Fengtai district | Dongfang hospital, Beijing electric power hospital, Beijing Fengtai hospital of intergrated traditional chinese and western medicine |
| Shijingshan district | Shijinshan hospital, Peking university Shougang hospital, Jingxi court of Beijing chaoyang hospital |
| Miyun district | Miyun hospital |
| Daxin district | Daxing hospital, Renhe hospital |
| Mentougou district | Beijing Jingmei group general hospital |
| Changping district | Changping hospital |
| Huairou district | Huairou first hospital |
| Fangshan district | Liangxiang hospital |
| Shunyi district | Shunyi hospital |
| Tongzhou district | Luhe hospital |
| Pinggu district | Pinggu hospital |
| Yanqing district | Yanqing hospital |

1. **Study subjects**

2.1 Inclusion criteria:

1. Patients presented to 35 sentinel hospitals in Beijing for acute respiratory tract infection (ARTI) between January 1, 2015 and December 31, 2020.
2. Upper respiratory tract infection (URTI) was defined as patients presented with fever and/or respiratory symptoms, e.g., cough, sputum production, and sore throat.
3. Community acquired pneumonia (CAP) was defined as patients presented with the evidence of CAP according to the guidelines for the diagnosis and treatment of adult community acquired pneumonia in China (released in 2016 by Respiratory Society of Chinese Medical Association) and the guidelines for management of community acquired pneumonia in children (released in 2013 by Pediatrics Society of Chinese Medical Association).
4. Patients or their guardians who agree to provide the written informed consent.

2.2 Exclusion criteria:

(a) Patients recently hospitalized for acute respiratory tract infection (<28 days for immunocompetent adult patients and <90 days for immunosuppressed adult patients, <7 days for immunocompetent child patients and <90 days for immunosuppressed child patients);

(b) Patients recently included in this study (within the previous 28 days);

(c) Patients with clear alternative diagnosis;

(d) Patients with some special medical conditions, e.g. newborn with ≤28 days old, pregnant or lactating woman, people with serious mental disorder.

1. **Sample Processing and Microbiological Investigations**

Total nucleic acids (RNA and DNA) were extracted from the respiratory specimens (Thermo Scientific™ KingFisher™ Flex Magnetic Particle Processors, Thermo Fisher).

The testing for a panel of 9 respiratory viruses, *Mycoplasma pneumoniae* (MP) and *Chlamydia pneumoniae*(CP), were performed on the extracted nucleic acids of all enrolled cases. The 16 respiratory virus-related indicators included inﬂuenza virus A and B (FLU A, FLU B), influenza virus AH1N1 2009 pandemic and AH3N2 (AH1N1, AH3N2), respiratory syncytial virus (RSV), parainﬂuenza virus 1, 2, 3, 4 (PIV 1, 2, 3, 4), adenovirus (AdV), human rhinovirus (HRV), human metapneumovirus (HMPV), human coronavirus 229E/NL63, OC43/HKU1 (CoV 229E/NL63, OC43/HKU1), human bocavirus (HBoV), human enterovirus (EV).

Whereas a panel of 13 respiratory bacteria, including *Stenotrophomonas maltophilia, Streptococcus pyogenes, Staphylococcus aureus, Klebsiella pneumoniae, Haemophilus influenzae, legionella pneumophila,* *Mycobacterium tuberculosis, Acinetobacter baumannii, Moraxella catarrhalis, Escherichia coli, Streptococcus pneumoniae, Pseudomonas aeruginosa* and *Pneumocystis jiroveci*, were tested only for those enrolled cases whose specimens of lower respiratory tract (sputum, pleural effusion, tracheal suction, bronchoalveolar lavage fluid. etc.) were available.

All viral and bacterial pathogens were tested with the use of the commercial real-time PCR based kits (Multiplex Combined Real-time PCR Detection Kit for Respiratory Viruses, Multiplex Combined Real-time PCR Detection Kit for Respiratory Bacteria, Jiangsu Uninovo Biological Technology Co. Ltd., China).
